# The Small Heat Shock Protein HSP25/27 (HspB1) Is Abundant in Cultured Astrocytes and Associated with Astrocytic Pathology in Progressive Supranuclear Palsy and Corticobasal Degeneration

**DOI:** 10.1155/2010/717520

**Published:** 2010-01-27

**Authors:** Lisa Schwarz, Grit Vollmer, Christiane Richter-Landsberg

**Affiliations:** Department of Biology, Molecular Neurobiology, University of Oldenburg, POB 2503, 26111 Oldenburg, Germany

## Abstract

Filamentous tau-positive protein inclusions in neurons and glia are prominent features of a number of neurodegenerative disorders termed tauopathies. These inclusions are further characterized by the presence of heat shock proteins (HSPs). The group of small HSPs, namely, HSP27 and *α*B-crystallin, interact with the cytoskeleton, bind to nonnative proteins, and prevent their aggregation after stress. To further investigate their contribution to neurodegenerative diseases, we have analyzed the association of HSP27 with pathological lesions of tauopathies. Microarray and immunoblot analysis revealed that HSP27 is enhanced at the mRNA and protein levels in affected brains, and that it is associated with astrocytic pathology. The upregulation of HSP27 in tauopathies with gial pathology implies distinct mechanisms for glial and neuronal cells. This was sustained by cell culture studies, demonstrating that the small HSPs are specifically and prominently expressed in unstressed astrocytes and not in neurons and in neurons remained at a rather low level even after stress situations.

## 1. Introduction

Filamentous protein inclusions are observed in a number of neurodegenerative diseases. They contain a cellular protein, such as *α*-synuclein or tau, and often a variety of heat shock proteins (HSPs) and ubiquitin in addition to cytoskeletal proteins [1–4]. HSPs acting as molecular chaperones refold damaged proteins and target irreversibly damaged proteins for degradation to the ubiquitin-proteasome system to prevent proteotoxic damage. Their presence in cellular inclusions indicates that they were unsuccessfully upregulated in an attempt to prevent protein denaturation and aggregation. Furthermore it indicates that stress situations, such as oxidative stress, heat stress, or stress induced by proteasomal impairment, contribute to the pathogenesis of the disorders [3].

Pathological inclusions containing the microtubule-associated protein tau in its hyperphosphorylated form are a characteristic hallmark of a class of both sporadic and familial disorders, termed tauopathies [1, 2, 4]. While in Alzheimer's disease (AD) tau aggregates preferentially are formed in neurons, in frontotemporal dementias, such as Pick's disease, progressive supranuclear palsy (PSP), and corticobasal degeneration (CBD), tau-positive inclusions are consistent features not only in neurons but also in glia [5–8]. Neuronal and glial filamentous lesions in PSP and CBD are composed of hyperphosphorylated tau with four microtubule binding repeats, that is, 4R-tau [9]. Tau is a microtubule-binding protein that is abundantly expressed in the central nervous system. It is important for microtubule assembly and stability, and in normal brain is mainly located in axons and not expressed in healthy astrocytes [10]. Data from our laboratory have demonstrated that it is also an important constitutent of the oligodendroglial cytoskeleton [7, 11]. Based on their cellular origin, in PSP and CBD tau-positive glial inclusions are classified as tau-positive astrocytes and oligodendroglial coiled bodies [8, 9, 12]. Astrocytic inclusions vary among the diseases; they do not form solid inclusion bodies but rather exhibit diffuse or fibrillary staining patterns. Tufted astrocytes and astrocytic plaques are typical for PSP and CBD, respectively, while tau-positive coiled bodies originating in oligodendrocytes can be found in both disorders [4, 12, 13]. 

The group of small HSPs with molecular weights in the range of 12–43 kDa is closely related [14, 15]. The best known representatives are *α*B-crystallin and HSP27, or its rat analogue HSP25. They are involved in a variety of cellular processes, particularly interact with cytoskeletal elements, are stress, inducible, display chaperone function and effectively bind to nonnative proteins, preventing the aggregation of proteins after stress [16, 17]. Cultured rat brain astrocytes constitutively express a high level of HSP25, which exerts protective effects against a variety of stress situations, while in oligodendrocytes *α*B-crystallin seems to be more important [18, 19]. In AD small HSPs have been found to be associated with astrocytes in senile plaques and not with neurons in neurofibrillary tangles [20]. *α*B-Crystallin immunoreactivity seemed to be specific to diseases with glial pathology [5, 21]. 

To further investigate the contribution of small HSPs to neurodegenerative diseases, we have analyzed the association of HSP27 with pathological lesions of PSP and CBD brains. Furthermore, since distinct pathogenic mechanisms and stress protein induction in neurons and astrocytes seem to contribute to tau pathology, we have compared HSP expression and upregulation in cultured neurons and astrocytes derived from rat brain. Immunoblot analysis demonstrates that HSP27 is upregulated in the brains of patients with PSP and CBD, and found in cell inclusions with astrocytic morphology. Furthermore, it is more prominently expressed in cultured astrocytes under normal and stress-induced conditions than in neurons.

## 2. Materials and Methods

### 2.1. Materials and Antibodies

Cell culture media were from Invitrogen (Grand Island, NY).

For Western Blot analysis or immunofluorescence the following antibodies were used: mAb anti-HSP70 (SPA-810), mAb anti-HSP/HSC70 (SPA-820), mAb anti-HSP60 (SPA-806), polyclonal anti-HSP40 (SPA-400), polyclonal anti-HSP32 (SPA-895), polyclonal anti-HSP25 (SPA-801), polyclonal anti-HSP27 (Spa-803), and mAb anti-*α*B-crystallin (SPA-222) from StressGen (Victoria, BC, Canada). Polyclonal anti-*α*-tubulin, mAb anti-*α*-tubulin, and mAb anti-GFAP were from Sigma (Taufkirchen, Germany). A mixture of mAbs anti-tau-14 and anti-tau-46 (both diluted 1 : 500) recognizing all Tau isoforms independently of phosphorylation, mAb PHF-1 (1 : 500) recognizing Tau phosphorylated at serine residues 396 and 404 and mAb Tau-1 specific for nonphosphorylated tau epitope located in amino acid residues 189–209 were used. The working dilution for Western Blot analysis was 1 : 1000 if not mentioned in parenthesis.

### 2.2. Sequential Biochemical Fractionation of Brain Tissue

**Figure 1:**
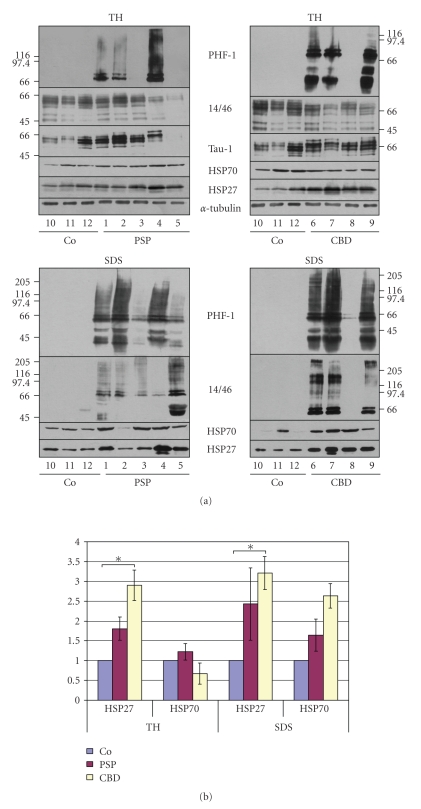
Biochemical analysis of tauopathy and unaffected control brains. (a) Representative images of total homogenate (TH) and SDS-fractions of globus pallidus from PSP (left) and CBD (right) brains are shown. Brain tissue (1 g/8 ml high salt buffer) was homogenized and subjected to sequential extraction (see materials and methods). Equal amounts of samples were subjected to SDS-PAGE and immunoblot procedure was carried out with antibodies against tau, HSP27, HSP70 and *α*-tubulin as indicated on the right. Co represent brain samples of unaffected controls. In (b) quantitative evaluation of the HSP27 and HSP70 blots shown in (a) and (b) was carried out using ImageQuant software. *α*-Tubulin from TH was used to normalize the data. Asterisk: *P* ≥ .05 according to *t*-test.

Brain tissue was obtained from the Center for Neurodegenerative Disease Research and the AD Center Core at the University of Philadelphia, School of Medicine. Brain tissue of frozen globus pallidus from PSP (*n* = 5), CBD (*n* = 4) and normal control (*n* = 3), was homogenized in 8 mL/g high salt buffer (HS) (weight/vol) (50 mmol/L Tris, pH 7.5, 750 mmol/L NaCl, 5 mmol/L EDTA; all buffers were supplemented with protease inhibitor cocktail (cOmplete, Roche Diagnostics, Mannheim, Germany) and phosphatase inhibitors PhosSTOP (Roche Diagnostics)) and centrifuged at 50,000 × g for 30 minutes at 4°C. Aliquots of the total homogenate (TH) were taken for immunoblot analysis. Pellets were extracted with 3 mL/g of HS buffer containing 1% Triton X-100 (HS-T). To remove myelin, pellets were homogenized in 500 *μ*L HS containing 1 mol/L sucrose. Floating myelin was discarded after centrifugation. The resulting pellets were homogenized in 2 mL/g of radioimmunoprecipitation assay (RIPA) buffer (50 mmol/L Tris, pH 8.0, 150 mmol/L NaCl, 5 mmol/L EDTA, 1% Nonidet P-40, 0.5% sodium deoxycholate, 0.1% sodium dodecyl sulfate (SDS)). Afterwards, pellets were extracted in 2 mL/g SDS buffer (62.5 mmol/L Tris, pH 6.8, 1 mmol/L EDTA, 0.1% *β*-mercaptoethanol, 2% SDS, 10% glycerol). The SDS-insoluble pellet was further extracted by formic acid, but did not reveal enough material for analysis. Fivefold concentrated sample buffer (312.5 mmol/L Tris, pH 6.8, 5 mmol/L EDTA, 0.5% *β*-mercaptoethanol, 10% SDS, 50% glycerol, 0.05% bromphenol blue) was added to TH, HS, HS-T, and RIPA, and all samples were heated to 100°C for 5 minutes. Equal volumes of samples from each fraction were loaded on 7.5 and 10% polyacrylamide gels. All fractions were analyzed by immunoblot procedure; see below. In the result section only the TH and SDS-fractions are shown (Figure 1). *α*-Tubulin of TH was used to normalize data.

### 2.3. Immunoblot Analysis

Cellular monolayers of control and treated cells were washed with phosphate buffered saline (PBS; 137 mM NaCl, 2.7 mM KCl, 1.47 mM KH_2_PO_4_, 8.4 mM Na_2_HPO_4_; pH 7.4) once, scraped off in sample buffer (125 mM Tris, pH 6.7, 1 mM EDTA, 1% *β*-mercaptoethanol, 10% glycerol), containing 2% SDS, and boiled for 10 minutes. Protein contents in the samples were determined according to Neuhoff et al. [22]. For immunoblotting, total cellular extracts (10–20 *μ*g protein per lane) were separated by one-dimensional SDS-PAGE using 7.5% or 10% polyacrylamide gels, and transferred to nitrocellulose membranes (Schleicher and Schuell, Dassel, Germany). The blots were saturated with TBS-T (20 mM Tris, pH 7.5, 136.8 mM NaCl, 0.1% v/v Tween 20) containing 5% dry milk and incubated with the individual antibodies overnight at 4°C. After washing, incubation with HRP-conjugated anti-mouse (Amersham Biosciences, Hercules, CA, USA; 1 : 3000) or anti-rabbit IgG (Biorad, Munich, Germany; 1 : 3000) was carried out for 1 hour, and blots were visualized by the enhanced chemiluminescence (ECL) procedure as described by the manufacturer (Amersham, Braunschweig, Germany). Quantitative evaluation of the immunoblots was carried out by densitometric scanning and ImageQuant software (Molecular Dynamics, Sunnyvale, CA, USA).

### 2.4. RNA Extraction and Microarray Analysis

To study the gene expression profiles total RNA was extracted of control and PSP globus pallidus brain tissue by Miltenyi Biotec (Cologne, Germany). Six human specific PIQOR microarrays (Miltenyi Biotec, Cologne, Germany) consisting of 1208 selected gene probes were used to generate an expression profile of mRNA in PSP brains versus control. For cDNA synthesis, RNA samples of two control brains and three PSP brains were pooled. cDNA synthesis and purification was carried out using the TSA-Labeling and Detection Kit (Perkin-Elmer, USA) according to the manufacturers instructions (see Perkin-Elmer, USA, Micromax TSA Labeling and Detection Kit for kits MPS521, MPS522 for details).

Hybridization and posthybridization were carried out using PIQOR Microarray Kit (Miltenyi Biotec, Cologne, Germany) according to the manufacturers instructions. Slides were scanned on a microarray scanner (GenePix 4000B, Axon Instruments, Foster City, USA). Acuity 4.0 (Molecular Devises, Califonia, USA) was used for signal quantification. Signals ≤0.5 refer to underexpressed genes, while signals ≥2 show overexpressed genes.

### 2.5. Immunohistochemistry

Tissue obtained at the time of autopsy was fixed in 10% formalin, paraffin-embedded and cut into 6 *μ*m thick sections. Following antigen retrieval using Vector unmasking solution formalin-fixed brain sections were analyzed by immunohistochemistry as described previously [23], using the avidin-biotin complex (ABC) method (Vectastain ABC kit, Vector Laboratories, Burlingame, CA). For double immunolabeling, sections were blocked in 5% ChemiBLOCKER (Chemicon, Millipore, Billerica, MA, USA) and 2% donkey serum in 0.1 M Tris, pH 7.6 supplemented with 0.1% Triton X-100 (TrisT) overnight at room temperature after antigen retrieval. First antibodies were diluted 1 : 100 in TrisT and incubated at room temperature overnight. After 6 washes for 30 minutes in 0.1 M Tris, pH 7.6, sections were incubated with secondary antibodies (1 : 100 in TrisT) (Jackson ImmunoResearch, West Grove, PA, USA) overnight at room temperature. Autofluorescence was blocked as described using Sudan Black B [24] and sections were mounted with Vectashield mounting medium (Vector Laboratories, Burlingame, CA, USA) containing 4′, 6-diamidino-2-phenylindole (DAPI). Digital images of immunohistochemical preparations were obtained using an Olympus BX 51 (Tokyo, Japan) microscope equipped with bright-field light sources with a digital camera, the DP71 (Olympus, Orangeburg, New York), and DP manager (Olympus). Fluorescent labelling was analysed using a Zeiss epifluorescence microscope (Oberkochen, Germany) equipped with a digital camera using a plan-neofluar objective (100× or 40× for overview images) or a Leica TCS SL confocal laser scanning microscope (Wetzlar, Germany).

### 2.6. Primary Neuronal Cell Culture

Single cell suspensions were prepared from cerebral hemispheres of 17-day-old rat embryos as previously described [25]. The tissue was homogenized in Eagles basal medium (BME), containing 0.5% heat-inactivated fetal calf serum (FCS). Cells were kept in serum-free medium supplemented with N2-Supplement (1 : 100; Invitrogen, Carlsbad, CA), L-glutamine (1 : 100; Invitrogen, Carlsbad, CA), 50 U/mL penicillin, and 50 *μ*g/mL streptomycin. Experiments were carried out with 7-day-old cultures or as indicated. Twice per week, one-half of the culture medium was exchanged.

### 2.7. Astrocyte Culture

Primary cultures of glial cells were prepared from the brains of 1-2-day-old Wistar rats and astrocytes were prepared from the flasks after 6–8 days as described previously [26]. Briefly, astrocytes were trypsinated from culture flasks, after removal of microglia and oligodendrocyte precursor cells, seeded on 10 cm culture dishes, and kept in DMEM supplemented with 10% fetal calf serum (FCS). 3 days before the experiment medium was changed to DMEM containing 0.5% FCS.

### 2.8. Heat Shock Treatment

Culture dishes were sealed with parafilm and immersed for 30 minutes in a water bath at 44°C, as described before [27]. For the indicated recovery, cells were put into the incubator. Control cells were sealed for 30 minutes but remained in the incubator.

### 2.9. Oxidative Stress

Cells were treated with hydrogen peroxide (100 *μ*M) for 30 minutes, the medium was replaced, and cells were put into the incubator for recovery as indicated.

### 2.10. Indirect Immunofluorescence

Cells were cultured on poly-L-lysine-coated glass coverslips (2.0 × 10^5^ cells per 35 mm dish) and subjected to heat shock or oxidative stress as indicated. After washing with PBS, cells were fixed with ice-cold methanol for 7 minutes. The coverslips were washed three times and incubated for 1 hour with anti-GFAP and anti-*α*-tubulin antibodies, followed by the appropriate secondary antibodies (1 : 100) (Jackson ImmunoResearch, West Grove, PA, USA), washed with PBS and mounted. Nuclei were stained by DAPI (1.5 *μ*g/mL) included in the mounting medium (Vectashield; Vector Laboratories, Burlingame, CA, USA). Fluorescent labeling was studied using a Zeiss epifluorescence microscope (Oberkochen, Germany) equipped with a digital camera using a plan-neofluar objective (100x).

## 3. Results

### 3.1. Increased Expression of Tau and HSP27 in Affected Brains of PSP and CBD Patients

Frozen tissue samples from the globus pallidus were obtained from 5 PSP and 4 CBD patients and from 3 control cases without neurological disease. The expression and solubility of tau was determined by sequentially extracting the brain tissue using buffers with increasing solubilization capabilities (see Methods section). The individual fractions were analyzed by immunoblot procedure using PHF-1 antibodies recognizing hyperphosphorylated tau, and tau-1 antibodies directed against nonphosphorylated tau, and a cocktail of t14 and t46 antibodies. Figure 1 shows the immunoblot of the total brain homgenates (TH) and the least soluble SDS-fraction. The majority of hyperphosphorylated tau was detected in the SDS fraction and was present in all PSP and CBD brain samples, although at different levels, thus confirming the pathology (Figure 1). Brain tissue was homogenized as weight per volume, and equal volumes of the subsequent individual fractions were separated by SDS-PAGE. Since *α*-tubulin was at approximately the same level in the TH of all brain samples, it was used to normalize data (Figure 1(b)). 

**Figure 2:**
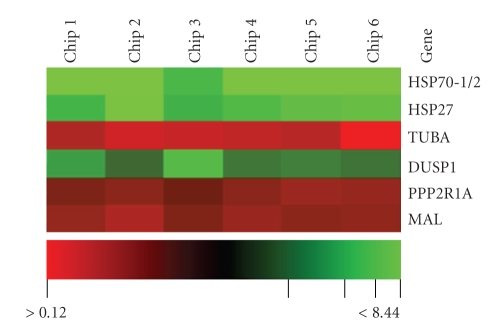
PIQOR microarray analysis of mRNA abundance in PSP versus normal control brains. Six chips were generated and statistically analyzed. Signals ≤0.5 (red) refer to underexpressed genes, while signals ≥2 (green) show overexpressed genes. The figure shows some genes out of 64 genes which are differentially regulated in PSP.: TUBA: *α*-tubulin; DUSP1: dual specifity protein phosphatase 1; PPP2R1A: serine/threonine protein phosphatase 2A; MAL: myelin and lymphocyte protein.

To study the expression profile of genes in normal and affected brains, RNA was isolated from PSP brain tissue and normal control tissue. RNA from 3 PSP and 2 normal control tissue samples was suitable and used for microarray analysis. Human specific PIQOR microarrays were used to analyze 1208 genes. Quantitative analysis revealed that 38 genes were repressed and 26 genes induced, including HSP27 and HSP70 (Figure 2). The induction of HSP27 mRNA was confirmed by RT-PCR analysis using the same RNA preparations (not shown).

This result prompted us to analyze the tissue extracts for the presence of HSP27 and HSP70 proteins (Figure 1(a)). Immunoblot analysis revealed that HSP27 was detectable in all fractions of the control and affected brain samples, and in comparison to the normal controls more abundant in the affected brains. In PSP brain extracts, HSP27 was elevated in the TH and most prominent in the least soluble SDS-fraction, and in CBD brains HSP27 was enriched in the TH and SDS-fraction. Also HSP70, as compared to control nonaffected brain tissues, was observable at an elevated level in the SDS-fraction of PSP and CBD brains (Figure 1(a)).

### 3.2. Increased HSP27 Immunoreactivity in Affected Brains

**Figure 3:**
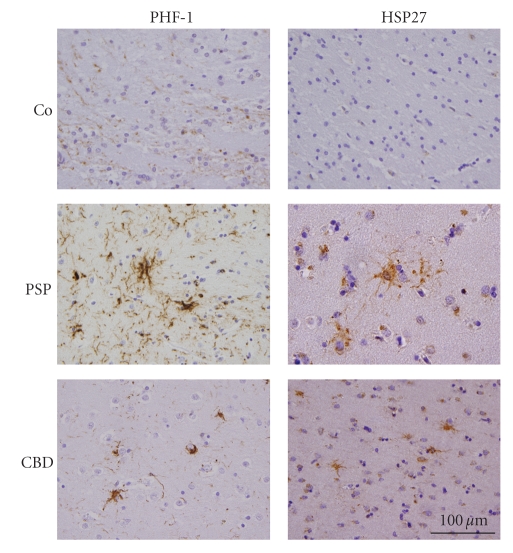
Expression of PHF-1 tau and HSP27 in globus pallidus of patients with PSP and CBD in comparison to unaffected control (Co). Adjacent sections were immunostained with PHF-1 (left) or HSP27 (right). PHF-1 staining demonstrates robust tau pathology in the affected brain regions. No pathology was detected in the brains of unaffected controls, while PSP and CBD brains show characteristic glial pathology and PHF-1 positive threads.

**Figure 4:**
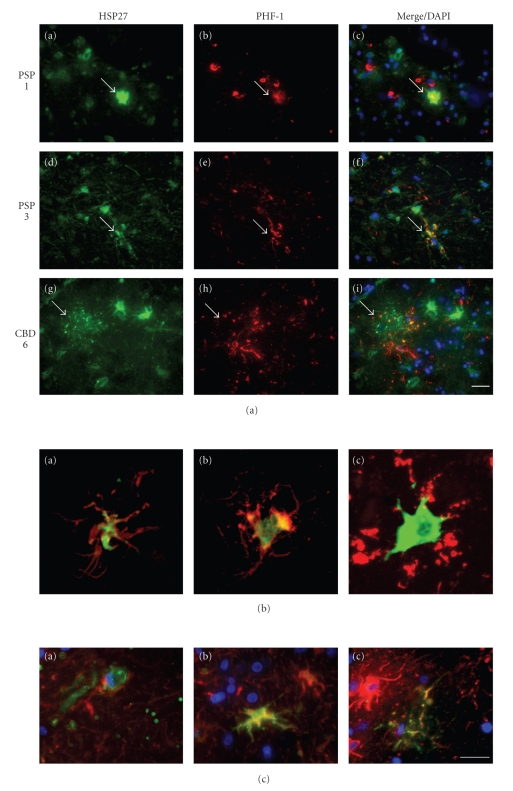
Colocalization of HSP27 with tau-immunoreactive inclusions. (b) Adjacent sections from affected brains, as indicated on the left, were double labelled for HSP27 (green) and PHF-1 tau (red). HSP27 colocalizes with a subset of astrocytic pathology. In PSP (a)–(f) astrocytic tufts positive for HSP27 and PHF-1 are seen (arrows) and in CBD (g)–(i), PHF-1 positive astrocytic plaques with HSP27 in the center are prominent (arrows). Scale bar, 25 *μ*m. Nuclei are stained with DAPI in the overlay images. (c) Confocal overlay images of representative cells from brain sections as depicted in (a) are shown (a,b, PSP patient 3; c, CBD patient 6). (d) Brain sections were double labelled for HSP27 (green) and GFAP (red). All cells positively stained for HSP27 were GFAP positive, demonstrating their astrocytic origin. Overlay images are shown for PSP patient 1 (a) and CBD patient 6 (b) and (c). Nuclei are stained with DAPI. Scale bar, 20 *μ*m.

Adjacent sections of paraffin-embedded globus pallidus tissue were prepared and immunostained with PHF-1 antibodies, to confirm tau-pathology, and antibodies against HSP27. As compared to the normal controls, a marked immunostaining of PHF-1 and HSP27 was observed in the affected brains (Figure 3). HSP27 staining was mainly associated with cells with astrocytic morphologies (Figure 3). Although PHF-1 positive coiled bodies originating in oligodendrocytes were present in the brain sections of PSP and CBD, we did not detect coiled bodies positively stained by HSP27 in the analyzed sections. This indicates that HSP27 pathology is mainly associated with astrocytes. This was further confirmed by double immunolabelling of brain sections using antibodies against HSP27 and PHF-1 (Figure 4). HSP27 immunoreactivity was present in numerous cells with astrocytic morphologies. Furthermore, differences in astrocytic pathology between PSP and CBD brain sections were observed. In PSP, PHF-1 positive cells resembling tufted astrocytes were often also positive for HSP27 (Figure 4, PSP patient 3). In CBD brain sections, PHF-1 positive astrocytic plaques were seen, which in the center contained HSP27 immunoreactivity (Figure 4, CBD patient 6). Furthermore, large astrocytes which strongly were labelled by antibodies against HSP27 containing PHF-1 immunoreactivity in the cellular extensions were typically observable in CBD brains (Figure 4, CBD patient 6). The colocalization of PHF-1 and HSP27 was further confirmed by confocal microscopy. The overlay images demonstrate that while HSP27 is mainly seen in the center of the cell throughout the cytoplasm, PHF-1 immunoreactivity is prominent in the periphery and the cellular extensions (Figure 4(b)). The data further show that not all HSP27-positive cells or plaques are also positive for PHF-1 but were colocalized in a subset of tau-immunoreactive inclusions (Figure 4). Also, even in brain samples with relatively little PHF-1 protein expression as demonstrated in the immunoblots (Figure 1, PSP 3 and CBD 8), HSP27 was abundantly expressed and in a subset of cells was found in colocalization with PHF-1. The astrocytic identity of the cells was corroborated by double immunofluorecent staining tissue sections using antibodies against GFAP (glial fibrillary acidic protein) and HSP27. Figure 4(c) demonstrates that cells expressing HSP27 are also positive for GFAP and thus represent astrocytes.

### 3.3. Constitutive Expression and Differential Upregulation of HSPs in Cultured Rat Brain Neurons and Astrocytes

**Figure 5:**
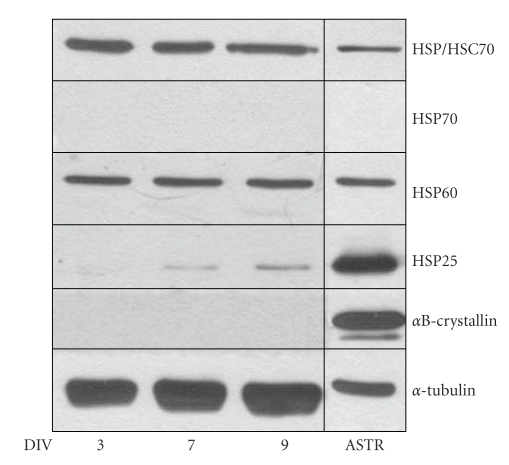
Cultured neurons and astrocytes constitutively express heat shock proteins at different levels. Cell lysates from astrocytes (ASTR) and neurons at the indicated days in vitro (DIV, 3, 7, 9) were prepared and equal amounts of proteins were subjected to immunoblot analysis using antibodies against various HSPs, as indicated on the right. Note that in comparison to neurons astrocytes constitutively express high amounts of HSP25 and *α*B-crystallin, while HSP60 and HSC 70 are similarly prominent in both cell types.

Previous data from our laboratory indicated that astrocytes in comparison to oligodendrocytes derived from the brains of newborn rats express a higher constitutive level of the small HSPs and thus might be protected from stress situations [18, 19]. Since tau-pathology is also prominent in neurons, and neuronal filamentous lesions and neuropil threads are found in PSP and CBD, we prepared cultures of rat cerebral neurons and astrocytes to compare the pattern of constitutively expressed HSPs. Cell extracts were subjected to immunoblot procedure using a panel of antibodies against several HSPs. Figure 5 demonstrates that astrocytes and neurons specifically differ in their abundance of the small HSPs, that is, HSP25 and *α*B-crystallin, while the higher molecular weight HSPs are similarly expressed in both cell types.

**Figure 6:**
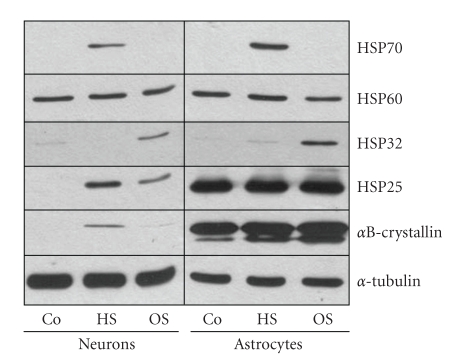
Differential stress responses of neurons and astrocytes. Neurons (7 div) and astrocytes were subjected to heat shock (HS; 44°C, 30 minutes) or oxidative stress (OS; 100 *μ*M H_2_O_2_) and cell lysates were prepared after a recovery period of 24 hours and equal amounts of proteins were subjected to immunoblot analysis, using antibodies against a variety of heat shock proteins, as indicated on the right. Note that in both neurons and astrocytes, HSP70 is heat-inducible, while HSP32 is only induced by oxidative stress. Generally, the induction of HSPs after stress is more pronounced in astrocytes than in neurons.

**Figure 7:**
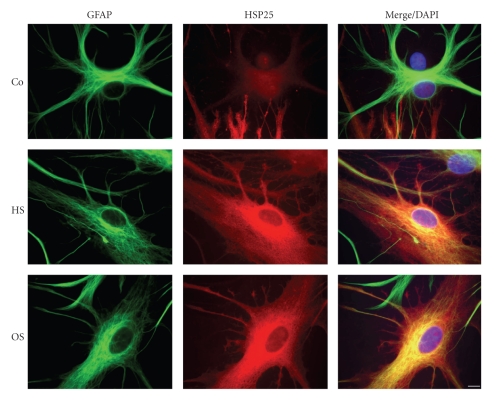
Cellular distribution of HSP25 in astrocytes. Astrocytes after heat shock (HS; 44°C, 30 minutes, 24 hours recovery) or oxidative stress (OS; H_2_O_2 _100 *μ*M, 30 minutes, 24 hours recovery) were subjected to indirect immunofluorescence staining. Co: untreated control. Double labelling was carried out using monoclonal anti-GFAP and polyclonal anti-HSP25 antibodies. Nuclei were stained with DAPI. Scale bar = 20 *μ*m.

Next we tested if neurons and astrocytes differentially react to heat and oxidative stress. Cells were subjected either to a heat shock (HS; 30 minutes, 44°C, 18 hours recovery) or to hydrogen peroxide (OS; 100 *μ*M, 30 minutes, 18 hours recovery). Immunoblot analysis reveals that HSP25 is upregulated in neurons after HS and OS, but under no condition is as abundantly expressed as in astrocytes (Figure 6). In neurons, *α*B-crystallin is inducible by HS but not detectable after OS, while in astrocytes it is enhanced after both stress conditions. In both cell types, HSP32 is induced after oxidative stress and HSP70 is induced only after heat stress, while HSP60 remains at the same level (Figure 6). The presence and upregulation of HSP25 in astrocytes after HS and OS was further confirmed by indirect immunofluorescence using antibodies against HSP25 and GFAP (glial fibrillary acidic protein). Figure 7 demonstrates that HSP25 is upregulated after HS and OS and distributed in the cell body and processes and partly is associated with glia filaments. Hence, these data show that HSPs are differentially expressed in neurons and glia, and that cells react to different stress situations by upregulation of different stress proteins. These data underline the functional significance of small HSPs in glia and point to the conclusion that cell type specific responses accompany pathological processes.

## 4. Discussion

In tauopathies such as AD, CBD, PSP, and Pick's disease, tau is the major constituent of intracellular inclusions, and the fact that these can occur not only in neurons but also in astrocytes and oligodendrocytes points out that glial cells contribute to the pathogenesis of neurodegenerative diseases [8]. Misfolded proteins assemble and accumulate before the onset of neurodegeneration, and it is likely that this is a mechanism aimed at removing denatured proteins to rescue the cells. When aggregates grow and enlarge, they may confer death signals and play a critical role in the pathogenesis of the diseases. It has been suggested that various types of inclusions may arise through common mechanisms. They contain components of the ubiquitin proteasome system and a variety of molecular chaperones [28]. Molecular chaperones provide a first line of defence against misfolded proteins and closely cooperate with the proteasomal machinery [29, 30]. HSP25/27 can confer resistance against apoptotic stimuli and might protect the cytoskeleton during stress [31, 32]. It undergoes dynamic assembly into large aggregates and oligomerization has been suggested to be required for chaperone function and substrate binding. The direct proof of a chaperone function of HSP27 in vivo is still missing, but evidence has accumulated that it contributes to an increased chaperone activity of the cells and binds unfolded proteins, which are then kept in a competent state to be refolded by HSP70 [33]. 

Here we examined the presence of HSP27 in PSP and CBD, representing tauopathies with abundant glial pathology [8, 12]. The data show that in affected brain tissue HSP27 is enhanced in comparison to the normal control, and a large proportion of HSP27 is only extractable with detergents disrupting the cytoskeleton, that is, 2% SDS, similarly to hyperphosphorylated tau. Also HSP70 is abundant in this fraction, and microarray analysis demonstrates that mRNA levels encoding HSP27 and HSP70 are enhanced in PSP brain samples. Immunohistochemical data further point to the fact that HSP27 is associated with cells displaying astrocytic morphologies, both in PSP and CBD brains, but we could not detect coiled bodies positively stained for HSP27. Double immunofluorescence labelling demonstrated that not all HSP27-positive cells displayed PHF-1 immunoreactivity, but astrocytic tufts positive for PHF-1 and HSP27 were prominent in PSP brain sections and PHF-1-positive astrocytic plaques with HSP27 immunoreactivity in the center were typically seen in CBD brain sections. Astrocytic tufts are prominent features of PSP brains and astrocytic plaques are characteristic in CBD [12]. Our data demonstrate that in both diseases a number of cells prominently express HSP27 without displaying tau pathology. This is also the case in brains of patients with a rather low level of PHF-1 protein expression. This might indicate that the stress response in astrocytes involving HSP27 upregulation precedes the formation of tau deposits and is attempted at protecting the cells against the protein aggregation process. 

Our data support a previous study, demonstrating that immunostaining for *α*B-crystallin and also HSP27, although to a much lesser extent, was increased in CBD, PSP, and FTDP-17 [21]. *α*B-Crystallin staining was specifically prominent in structures resembling coiled bodies in CBD and FTDP-17. In the three PSP cases analyzed, which displayed only mild tau pathology, also only mild *α*B-crystallin immunoreactivity was observable [21]. HSP70 immunostaining was not increased in both affected and unaffected brain regions, and HSP27 immunostaining was demonstrated predominantly in glial cells in the neocortex of all three pathologies, but this was not further persued biochemically in this previous study [21].

Thus, the upregulation of the small HSPs seems to be specific for glial pathology, and implies distinct pathogenic mechanisms for glial and neuronal cells. This is supported by our data, demonstrating that cultured neurons and astrocytes have a different set or amount of constitutively expressed HSPs and that the small HSPs, *α*B-crystallin and HSP25, are specifically and prominently expressed in unstressed astrocytes. Even after stress situations the levels of small HSPs in neurons remain low. Similarly, oligodendrocytes have a low constitutive level of both small HSPs, but respond to oxidative and proteasomal stress by prominent induction of *α*B-crystallin, while HSP25 is mainly induced after heat stress [18, 27]. Astrocytes are specifically resistant against a variety of stress situations and our recent data demonstrate that they are protected by their high constitutive level of HSP25 [19, 32]. Downregulation of HSP25 by siRNA approach caused actin filament breakdown in control cells in the absence of stress stimuli and sensitized the cells against oxidative and proteasomal stress [19]. Hence cell-type and stress-specific regulation is observable in cell culture and likely involved in disease progression. 

The question remains, why small HSPs are associated with the insoluble protein fractions of stressed cells and affected brains. Indeed, although small HSPs may prevent protein aggregation by forming soluble complexes with aggregation prone substrate proteins, they are often found in insoluble detergent-resistant fractions [19, 21, 34]. Cell culture studies have revealed that at an early state of pathogenesis small aggregates and aggresomes are formed, these are aimed at shielding the remaining part of the cell body from unwanted interactions with misfolded proteins [32, 35, 36]. In glial cells, aggresomes are formed around the MTOC and small HSPs and ubiquitin are recruited to the growing inclusions as a major constituent [19, 37, 38]. When cells are overloaded with nonnative substrates, small HSPs coaggregate with nonnative proteins and are tightly associated with and might surround inclusion bodies. This situation is specifically promoted in stress situations, such as oxidative stress or stress exerted by proteasomal inhibition. 

To summarize, small HSPs seem to be particular important in neurological diseases with glial pathology. They are prominent constitutents in inclusion bodies originating in astrocytes and oligodendrocytes. These are characteristic features of CNS diseases with cytoskeletal abnormalities. Although small HSPs can specifically interact with cytoskeletal components and might preserve cell shape and integrity, chronic overexpression, instead of protecting the cells, might further contribute to cell damage and disease progression.
